# The unity hypothesis revisited: can the male/female incongruent McGurk effect be disrupted by familiarization and priming?

**DOI:** 10.3389/fpsyg.2023.1106562

**Published:** 2023-08-29

**Authors:** Kennis S. T. Ma, Jan W. H. Schnupp

**Affiliations:** ^1^The School of Psychology & Counselling, The Open University (UK), Milton Keynes, United Kingdom; ^2^Department of Neuroscience, City University of Hong Kong, Kowloon, Hong Kong SAR, China

**Keywords:** McGurk, multisensory integration, top-down, speech perception, multi-modal

## Abstract

The unity assumption hypothesis contends that higher-level factors, such as a perceiver’s belief and prior experience, modulate multisensory integration. The McGurk illusion exemplifies such integration. When a visual velar consonant /ga/ is dubbed with an auditory bilabial /ba/, listeners unify the discrepant signals with knowledge that open lips cannot produce /ba/ and a fusion percept /da/ is perceived. Previous research claimed to have falsified the unity assumption hypothesis by demonstrating the McGurk effect occurs even when a face is dubbed with a voice of the opposite sex, and thus violates expectations from prior experience. But perhaps stronger counter-evidence is needed to prevent perceptual unity than just an apparent incongruence between unfamiliar faces and voices. Here we investigated whether the McGurk illusion with male/female incongruent stimuli can be disrupted by familiarization and priming with an appropriate pairing of face and voice. In an online experiment, the susceptibility of participants to the McGurk illusion was tested with stimuli containing either a male or female face with a voice of incongruent gender. The number of times participants experienced a McGurk illusion was measured before and after a familiarization block, which familiarized them with the true pairings of face and voice. After familiarization and priming, the susceptibility to the McGurk effects decreased significantly on average. The findings support the notion that unity assumptions modulate intersensory bias, and confirm and extend previous studies using male/female incongruent McGurk stimuli.

## 1. Introduction

Audition is the dominant modality in speech perception for normally hearing individuals, and all the information necessary for perceiving speech is contained within the acoustic signals. Nevertheless, individuals do often combine visual “lipreading” cues with auditory inputs to perceive a “best-fit utterance” (Ma et al., 2009; Schnupp et al., 2011) that takes advantage of all available information channels to increase perceptual accuracy. The McGurk effect is a compelling demonstration that speech perception is an inter-modal, rather than a unimodal phenomenon, as it reveals a strong visual bias on auditory perception. In their classic study, McGurk and MacDonald (1976) constructed auditory–visual (AV) mismatched speech signals by dubbing a voice producing one consonant, such as /b/, over a mouth articulating a discrepant consonant, such as /g/. This induced perceivers to experience an auditory illusion, where the heard consonant may match neither the visual nor the auditory one, but instead a “fusion” consonant is perceived. For instance, many observers report hearing a /d/ when the visual velar phoneme /g/ is dubbed with an auditory bilabial phoneme /b/ (McGurk and MacDonald, 1976). In other instances, vision may appear to dominate. For example, when a video of a mouth articulating a phoneme /d/ is dubbed with an auditory phoneme /b/, perceivers often report hearing the visual /d/ rather than the acoustic /b/ (Tiippana, 2014). Such visual bias effects have been replicated many times, and the phenomenon is overall very robust (Green et al., 1991). However, a wealth of evidence also suggests that observers may differ greatly, in a stimulus-dependent manner, in how susceptible they are to visual influences on the auditory perception of a given syllable. Thus, any one observer tested repeatedly with a given audio-visual clip will perceive a McGurk illusion “either almost always or almost never,” but the same observer may respond differently to a different clip, and the proportion of observers who fail to perceive an illusion for any one clip is often substantial (Mallick et al., 2015; Stropahl et al., 2017).

Welch and Warren (1980) proposed a model of multisensory interactions, outlining conditions under which intersensory bias occurs when the brain attempts to maintain coherent perception in the face of discrepant signals from more than one modality. According to their model, the perceptual outcome of whether discrepant sensory inputs are treated as a single multisensory object or event is determined by stimulus-driven and higher-level factors. They put forward the “unity assumption” hypothesis, which proposes that a perceiver’s belief and expectation (either conscious or unconscious) determine whether two or more sensory signals are treated as a single multisensory object or event or not. According to this theory, McGurk effects should only arise if observers can plausibly make the unity assumption that the lip movement that is seen and the syllable that is heard both originate from one and the same vocal source. Formal models that can describe this type of cognitive process quantitatively are sometimes referred to as “causal inference models” (that is, the observer infers how likely it is that the sensory information received from different sensory streams originates from a single, common cause or multiple different causes, and causal inference models have been applied successfully to quantitatively describe sensory integration in spatial localization tasks (Körding et al., 2007) as well as in the context of the McGurk illusion (Magnotti and Beauchamp, 2017; Magnotti et al., 2018). In such quantitative approaches, the top-down prior expectations that there is only a single source can be conceptualized in a Bayesian framework, where they are thought of as a “common source prior” (Sato et al., 2007). That prior strongly influences whether multimodal stimuli are integrated or segregated, and it is shaped by experience. That such prior expectations can influence multisensory integration by changing top-down assumptions has been shown in an experiment by Gau and Noppeney (2016), in which they delivered stimuli in blocks that created different, phonetically matched or mismatched contexts. This affected the susceptibility of participants to the McGurk illusion. Imaging data recorded in the same study furthermore suggested that the inferior frontal sulcus may play an important role in judging cross-modal congruence.

However, Green et al. (1991) published results of experiments some time ago which used variants of the original McGurk paradigm, and which appeared to contradict Welch and Warren’s unity assumption hypothesis. Their experiments sought to investigate how robust the McGurk effect is if the visual and auditory stimuli are made “incongruent” in a manner that should make the unity assumption highly implausible. One example of such incongruence involves dubbing videos of a woman mouthing syllables for a McGurk effect with sound recording of a man pronouncing the same syllables (As an aside, Green et al. wrote at a time when the word “gender” was commonly used as an alternative term for biological sex, and they referred to this type of stimulus as “gender incongruent.” Given contemporary preferences to reserve the word “gender” for the discussion of issues of psychological or societal gender identity which are not relevant in this context, we shall refer to this type of stimulus as male/female incongruent here). Given that the adult human vocal tract is quite strongly sexually dimorphic, this produces highly incongruent percepts. Testosterone bursts during puberty trigger a lengthening of the male vocal tract, which causes adult male voices to be typically not just lower in pitch, but also to have lower formant frequencies than those of females. Vocal tract length is not under voluntary control, and the distribution of formant frequencies in a voice provides a strong cue to the biological sex of the speaker which is independent from voice pitch. People learn to pick up on these acoustic cues during childhood, and become highly proficient in distinguishing male and female voices by 10 years of age (Nagels et al., 2020). Consequently, adult observers who are shown such male/female incongruent AV stimuli will find the disparity between voice and face very obvious, and this should give a strong cue that the face cannot belong to the voice, and the visual evidence should therefore be ignored. Yet despite the salient incongruence in the sexual characteristics of voice and face, many participants in this study did perceive a McGurk illusion. Green et al. (1991) argued that this falsifies Welch & Warren’s theory.

However, it is perhaps premature to assert that male/female incongruence must necessarily prevent perception of unity. For example, in male/female incongruent McGurk stimuli, there is still a precise temporal synchrony between the visual and auditory stimuli. Such precise synchronization is very unlikely to arise by mere coincidence. Indeed, synchronization (that is, a high degree of temporal correlation), has been shown to act as a strong AV binding cue (Parise et al., 2012). This may induce participants to perceive a single bimodal stimulus which remains unified despite its confusing sexual characteristics, rather than a separate face and a separate voice which just happen to be tightly synchronized by chance. In the terminology of Welch and Warren (1980), one could argue that the precise temporal synchronization in the dubbed videos creates a “highly compelling” situation in which the assumption of unity remains sufficiently strong despite the peculiar voice given the face, and “intersensory bias” can change the heard /b/ to /d/. Welch & Warren never developed a mathematical framework for their unity assumption hypothesis, and it is likely an oversimplification to conceptualize the issues surrounding these phenomena as strictly binary, such that the unity assumption must either hold or not, and that particular AV mismatched consonant must be either heard as fused or not. In contrast, Bayesian conceptions of the McGurk effect (Sato et al., 2007) would allow us to think of the likelihood of unity as varying along a continuum as a function of “posterior” sensory evidence and prior knowledge, and a recent study by Getz and Toscano (2021) also raises the issue that the forced classification of the perceived AV consonants as “fused” or “unfused” can be a rather too limited description that fails to capture a wider underlying range of possible perceptions, which may result from complex interactions of stimulus properties and individual differences. Rather than positing that the unity assumption must either apply or not, it may be better to conclude from Green et al.’s (1991) study that male/female incongruence alone typically only slightly reduces the strength of the unity assumption if other factors such as temporal or spatial coincidence still support the unity assumption.

Of course, in that framework, the “posterior probability” that the unity assumption applies may further decline in the minds of participants if additional evidence and prior knowledge argue against it. Some early evidence that this may be so came from a study by Walker et al. (1995), who sought to investigate whether the susceptibility of McGurk effect in male/female incongruent stimuli decreases when participants knew the speaker. The logic here is that the incongruent pairing of face and voice in a male/female incongruent McGurk stimulus should be particularly striking if the face seen in that stimulus is a familiar one, and the observers know from experience that the voice “does not fit,” not just because it is clearly male/female incongruent, but also because it is not the voice that belongs to a face of a person they know and recognize. Familiarity with a person can thus increase an observer’s expectations and thereby amplify the incongruence perceived when the video of a familiar face is synchronized with a “wrong” voice. To investigate whether familiarity matters, Walker et al. presented, in each trial, a familiar or unfamiliar face that was paired with a voice that was either congruent (same person) or incongruent (different person of either the same or opposite sex). They found that participants who saw the unfamiliar faces showed no differences in McGurk effect, regardless of whether faces were paired with voices of the same or opposite sex. This was in line with Green et al.’s (1991) findings. However, when the face of a familiar person was combined with a different person’s voice (either same or opposite sex), the McGurk illusion decreased, as might be predicted by Welch and Warren’s theory.

The Walker et al. (1995) study thus indicates that familiarity with the stimuli may generate a set of “priors,” that can modulate the strength of the unity hypothesis in certain contexts. We were interested in investigating whether we could replicate their findings with only very modest amounts of familiarity with the stimuli, which can be delivered over a small amount of time. Walker et al. did not use a within-participant design, but rather compared cohorts of participants who were familiar with the faces in the stimuli with cohorts that were not. This could be problematic given that it is well established that individuals vary widely in how susceptible they are to the McGurk effect. Indeed, some individuals appear insensitive to the McGurk effect, and perceive it 0% of the time when tested with a particular set of AV stimuli, while other participants perceive it 100% of the time when tested with the same stimuli. Other participants may lie somewhere in between, perceiving the McGurk illusion sometimes but not always (Mallick et al., 2015). Furthermore, the magnitude of the McGurk effect is stimulus-dependent, in the sense that the proportion of fused syllables that is perceived by any one particular participant may vary in idiosyncratic ways depending on which faces or voices are used to construct the stimuli (Stropahl et al., 2017). Another factor that has been shown to influence the likelihood of perceiving a McGurk illusion is whether the mismatched audio-visual syllable is presented only once or repeated several times in quick succession (Magnotti et al., 2018). A recent study by Getz and Toscano (2021) contains an excellent overview of the many factors that have been shown to create individual differences in the susceptibility to the McGurk illusion. These range from age to linguistic background to mental health status, which could create confounds in studies that happen to have unbalanced cohorts with respect to such factors.

In the current study we sought to establish whether the key findings by Walker et al. (1995) can be replicated under a within-participant experimental design, which can cancel out potential problems stemming from the high degree of variability between individuals by measuring the strength of the male/female incongruent McGurk effect both before and after a standardized amount of familiarization and priming. Stimuli for this study were created from a subset of the validated corpus of video and audio clips for McGurk effect research known as the “Oldenburg Audio Visual Speech” (OLAVS) stimuli (Stropahl et al., 2017). The hypothesis was that the same gender male/female stimuli would evoke fewer McGurk illusions after the participants had been familiarized with the “true,” gender congruent pairings of the voices and faces that had been recombined into male/female incongruent dubs in an initial experimental block. Constraints that influenced our design choices were that the experiment had to be delivered entirely online, due to social distancing restrictions in force at the time, and the experiment had to be short as we were relying entirely on the goodwill of volunteer participants who received no compensation for their time. To maximize the chances of seeing an effect in an experiment that took only about half an hour to complete, and therefore allowed only little time for familiarization training and no time for consolidation, we also reminded participants of the appropriate pairing between face and voice by priming with a male/female congruent example just prior to a re-test with the male/female incongruent stimuli. This combined familiarization plus priming approach proved effective. Within the time and resource constraints of this study, we were, however, not able to determine whether familiarization or priming alone might have been sufficient to produce the observed effects.

## 2. Method and materials

### 2.1. Stimuli

The stimuli for the experiment were constructed from 6 soundtracks and 6 videos which were adapted from a subset of the corpus of video and audio clips for McGurk effect research known as the “Oldenburg Audio Visual Speech Stimuli (OLAVS)” (Stropahl et al., 2017). The OLAVS corpus contains a set of 64 temporally aligned audio and video clips of 8 speakers (4 female, 4 male) uttering the syllables /ba/, /da/, /ga/, /ka/, /ma/, /na/, /pa/ and /ta/. All audio and video clips are ~2 s long and arranged such that the onset of the syllable in each occurs after about 750 (+/−10) ms. The original video clips in the corpus were 25 fps mpegs, and the audio recordings were uncompressed 24 bit WAV files with a sample rate of 48 kHz. These were designed by Stropahl et al. (2017) to be used for the easy creation of McGurk illusion videos simply by simultaneously presenting a desired combination of audio and video clips.

For our experiment here we selected a subset of the clips from the OLAVS corpus and recombined them as needed (see further details below). To allow rapid online delivery in our experiment, we processed the selected clips using the command line media file editing tool ffmpeg,^1^ combining the sound and video in a single MP4 file, after cropping and downsampling the videos to 360×360 pixels. Throughout, all audio signals were presented diotically, with the same signal sent to the left and right audio channels. Custom JavaScript code was prepared to embed the video clips into a Qualtrics online survey, which was used to allow participants to perform the experiment using their own computers and standard web browsers.

For the purposes of this experiment, the sound and video clips were combined into audio-visual stimuli which could have disparities along two separate features: there could be a sex disparity between the face in the video and the voice in the audio (e.g., a male face dubbed with a female voice), or there could be a phonetic disparity between the onset consonants in the visual and auditory parts of the stimuli (e.g., /ba/ dubbed with /ga/), or both. For clarity, we shall refer to sex disparity as “male/female incongruence” and to phonetic disparities as “AV mismatch” throughout the rest of the manuscript.

The stimuli for 12 practice and 98 experimental trials were constructed by combining voices and faces of the /ba/, /ga/ and /da/ syllable recordings from one male and one female actor from the OLAVS set [identified in Stropahl et al. (2017) as “TK05” and “TK01”], as required. Phonetically matched (A: /ba/ V: /ba/ or A: /ga /V: /ga/) and mismatched (A: /ba/ V: /ga/) combinations were produced. The mismatched combinations served to test whether, and how often, participants experienced a McGurk fusion percept /da/, while the phonetically matched stimuli provided a simple but effective control measure for assessing data quality. Correctly identifying the unambiguous syllable presented during phonetically matched stimuli ought to be easy, and frequent failures to identify these syllables correctly give a strong indication that a participant was inattentive or for other reasons unable to perform the task adequately, and that their data needed to be excluded.

A list of instructions, an informed consent form, and a debriefing page, were also incorporated into the Qualtrics survey used to conduct the experiment.

### 2.2. Paradigm

A repeated-measures (within-subject) factorial ANOVA design was used to investigate the effects of AV match (Independent variable 1) and familiarization and priming (Independent variable 2) on the susceptibility to McGurk illusion (Dependent variable). Both independent variables had two levels (phonetically matched or mismatched; before or after familiarization and priming). Male/female incongruent AV combinations were used to measure the strength of the male/female incongruent McGurk effect, first at baseline in Block 1, and then again after familiarization and priming in Block 3. Male/female congruent combinations were used during familiarization with the “true” pairing of face and voice in Block 2, and for priming stimuli in Block 3 (see Paradigm below for further details). Trials were randomized within blocks so participants could not predict which syllable came next. The dependent variable was the number of times participants report hearing the fusion sound/da/in mismatched stimuli. In this design, we reject the null hypothesis “familiarization and priming has no effect on the strength of the male/female incongruent McGurk illusion” if the ANOVA shows a significant interaction between the two independent variables, as that would demonstrate that the rate at which AV-mismatched stimuli evoke fusion percepts depends on familiarization and priming. Counterbalancing was used to randomly allocate half of the participants to experience incongruent male voice/female face stimuli, and the other half to the opposite pairing.

Motivated by social distancing guidelines during the COVID-19 pandemic, the experimental sessions were conducted online with no experimenters present. Participants used their own computers and headphones to access an online survey implemented on the Qualtrics platform, which guided them through the experiment. For the trials, the experimental video and audio clips were embedded into the online survey questions using custom written JavaScript code. Informed consent was obtained before each experiment. Participants were informed of the nature of the experiment, and were instructed to watch trial videos attentively in a quiet room with no distractions. While this type of online format afforded the experimenters little ability to standardize stimulus presentation or monitor the experiment, these issues were very effectively mitigated by the within-subject design of the study. Individual differences in experimental settings, which arise as each participant uses their own consumer grade hardware for stimulus presentation in a space of their own choosing, can be factored out given that each subject serves as their own control. Furthermore, this online format also increases the ecological validity of the study; we were able to observe a statistically highly significant effect (see below) in data collected from participants who each took part in the experiment in their own, familiar environment, and using their own equipment, rather than having to perform the task in what would for many of them be a highly unnatural laboratory environment. If we can observe a clearly significant effect despite the high intra-subject variability we expect given not just their intrinsic individual differences but also the differences in equipment and setting, then this would increase our confidence that the results may have general validity that extends beyond rigid laboratory conditions.

Before the experiment, participants answered a questionnaire asking for voluntary information about their age and gender, and asked them to confirm self-reported normal hearing and normal or corrected-to-normal vision. To preserve the participants anonymity, no further identifying personal information was collected. Before the start of the experiment proper, participants were guided through 12 practice trials to familiarize them with the trial format and user interface. The online survey then guided the participants through the three experimental blocks. Block 1 measured the baseline rate with which participants experience a McGurk illusion when tested with our male/female incongruent AV-mismatched stimuli, Block 2 then familiarized the participants with the actual (same sex), voice for the face they encountered in Block 1 by engaging them in a selective attention listening task, and finally, Block 3 again measured the rate of McGurk illusions with the stimuli used in Block 1, but this time with “priming” which frequently reminded the participants of the true face-voice pairings.

**Figure 1:**
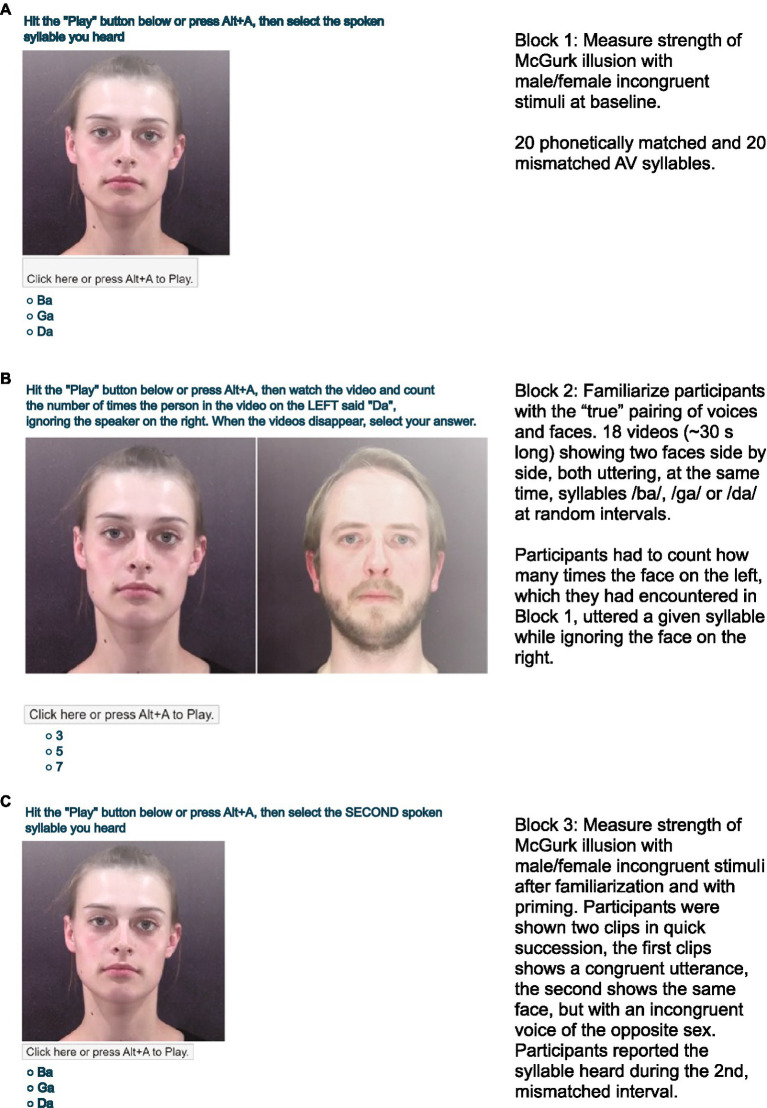
Screenshots illustrating the stimuli presented to the participants during each of the 3 blocks. The experiment first measured the strength of the male/female incongruent McGurk illusion in each participant **(A)**, then engaged the participants in a task that encouraged them to form a correct association between the faces and voices used in the experiment **(B)**, and finally retested the participants with male/female incongruent stimuli, but in this final, post-familiarization block participants were additionally reminded of the correct face-voice pairing by priming just prior to the presentation of each test stimulus **(C)**.

In Block 1, to measure participants’ baseline performance with male/female incongruent stimuli, participants were instructed to watch randomly interleaved presentations of 20 phonetically matched (10 A: /ba/ V: /ba/ and 10 A: /ga/ V: /ga/) and 20 mismatched AV (A: /ba/ V: /ga/) syllables (Figure 1A). In each trial, a voice producing /ba/ or /ga/ was dubbed with a ~2 s long, male/female incongruent face articulating either /ba/ or /ga/. Participants reported whether they heard /ba/, /ga/ or /da/ using their computer mouse or keyboard (A three-alternative forced choice design). Percepts of /da/ were counted as fusion percepts.

In Block 2, to familiarize participants with the true, same-sex pairings of the voices and faces that had been incongruently recombined in Block 1, participants were engaged in an observation task in which they had to attend to one speaker without getting distracted by a second speaker. To provide a strong perceptual pairing of the face from Block 1 with its own, true voice, we designed a stimulus in which two speakers were simultaneously present, one being the face of Block 1 speaking with its own voice, the other the voice of Block 1 paired with its own face. Both speakers were uttering syllables at independent random intervals, with possible temporal overlap, that is, one face might start uttering a syllable before an utterance by the other face had finished. This created a “cocktail party scenario,” where the presence of multiple speakers places high attentional demands on the observer, so that syllables uttered by an attended speaker can be identified without interference from the syllables uttered by the unattended speaker. We reasoned that such a “cocktail party” scenario might be particularly effective in making observers attend to, and thereby learn, which voice really belongs to each face. Participants watched 17 video clips, each ~30 s long, showing the two faces side by side (Figure 1B), each uttering syllables /ba/, /ga/ or /da/ a random number of times, at randomized intervals. The number of times each syllable was presented was a random permutation of the set {3, 5, 7}, and the order of presentations was randomized independently for the left and right speakers. Participants were not briefed on the algorithm for generating the random syllable sequences. The face on the left was always the face the participants had encountered in Block 1, while the face on the right was the true face belonging to the voice encountered in Block 1. All utterances by both faces were therefore male/female congruent, and they were also AV matched throughout Block 2. Participants initiated each trial by clicking the screen element labeled “Click Here or press Alt-A to Play.” The face on the left produced its first syllable at a time chosen uniformly at random from the range [100, 300] ms, and thereafter produced syllables at random intervals chosen uniformly from the range [1,500, 1,900] ms. The face on the right produced its first syllable at a random time chosen uniformly from the range [400, 600] ms and subsequent syllables at intervals also chosen from a [1,500, 1,900] ms range. This ensured that the left face started speaking first, but thereafter both faces spoke at independently randomized time intervals. Participants were instructed to count how many times the face on the left uttered either /ba/, /ga/ or /da/, while ignoring the face on the right, and report their count at the end of each trial. This counting task is very difficult, as it makes high demands not just on selective attention but also on working memory, and many participants failed to get even a single trial correct. Nevertheless, performance at this task is greatly aided if participants strongly associate the Block 1 face with its true voice, and dissociate it from the incongruent Block 1 voice, as this would allow interference from the face on the right to be more easily ignored. The participants received plenty of cues during this block to help them form the correct association between faces and voices, not only because there is now a male/female congruence that meets a typical observer’s expectations, but also because, despite the occasional temporal overlap in syllables uttered by the left and right speaker, there is a nevertheless a tight temporal AV synchrony only between the male/female congruent AV syllables. Consequently, we assumed that merely attempting this task and watching the two speakers speak independently side by side ought to facilitate the formation of strong associations between each face with its “true” voice.

Block 3 re-measured the strength of the McGurk effect with the same male/female incongruent stimuli from Block 1. However, to remind the participants of the familiarization in Block 2, in each trial, participants were shown two clips in quick succession. First, a ~2 s male/female congruent, AV-matched stimulus chosen at random from the syllables /ba/ and /ga/ reminded participants of the face that actually belonged to the voice in the upcoming target (priming). This was followed immediately by a male/female incongruent target clip identical to the ones used in Block 1. Participants performed 40 trails in this block with phonetically matched or mismatched AV target stimuli, and were prompted to indicate whether they heard /ba/, /ga/or/ da/ during the second clip (Figure 1C). Presenting a priming stimulus before each target in Block 3 does introduce a confound: our data do not allow us to assess the extent to which any observed changes in the participants’ susceptibility to the male/female incongruent McGurk illusion was attributable to the familiarization gained on Block 2, or the presence of the prime in Block 3, respectively. While it would in principle have been possible to disambiguate this confound through the introduction of additional, unprimed conditions in Block 3, doing so would have considerably increased the total duration of the experiment, and we feared that this would have resulted in impracticably high drop-out rates among our unpaid, anonymous volunteer participants. The present experimental design was a compromise, which sacrificed the ability to dissociate priming from training effects in order to maximize the likely overall effect size by combining familiarization and priming, as well as keeping statistical power high by keeping drop out rates low in an experiment that was as short as possible.

The entire experiment took ~25 min. At the end of the experiment, the purpose of the experiment was explained in greater detail with a debriefing sheet incorporated into the Qualtric online survey.

All procedures were conducted in compliance with the British Psychological Society’s Code of Human Research ethics and approved by the DE300 ethical review board of the Open University. Participants gave informed consent at the beginning of the online survey, and were given the opportunity to ask any questions by email. A personal number was issued to each participant to enable them to request the withdrawal of their data. None of the participants availed themselves of that option.

### 2.3. Data analysis

The participants’ 3-alternative forced choice responses (/ba/, /ga/ or /da/) to the McGurk stimuli were coded as a “fusion percept” variable. Participants reporting that they heard the syllable /da/ was scored as a “fusion,” and hence an incidence of a McGurk illusion. Other reported auditory percepts (/ba/ or /ga/) were scored as unfused. The main objective of this study was to determine whether the proportion of fusion responses to male/female incongruent stimuli decreases after familiarization and priming with the correct voice and face pairings. The within-subject design allowed us to examine this question by comparing “fusion” scores at baseline (Block 1) with those obtained after familiarization and priming (Block 3). Because scoring fusion percepts only as outcome variable neglects the possibility that visual influences might also manifest as visual dominance responses (that is, a A: /ba/ V: /ga/ stimulus might be reported as being heard as /ga/ if lipreading dominates the perception on a particular trial) we also computed a “total AV interactions score,” which sums the number of /da/ and /ga/ responses reported by each participant.

Two statistical tests were used to assess the statistical significance of these results. A 2 × 2 repeated measure factorial ANOVA was performed with two independent variables: “AV match” and “Block.” The “AV match” variable had two levels: either mismatch between the auditory and visual utterance for McGurk stimuli, or match for control stimuli. The “Block” variable also had two levels, either “Block 1 baseline” or “Block 3 primed”). In the ANOVA model, the AV match term therefore measures the effect size of the McGurk illusion (*a priori* we expect “fusion” percepts to be quite common for AV mismatched, but not matched, stimuli), and the AV match × Block interaction term measures how much the McGurk illusion (AV match effect) is altered by the “treatments” (familiarization and priming) that distinguish Blocks 1 and 3. A statistically significant AV match × Block interaction can therefore be interpreted as evidence that familiarization and priming change the strength of the McGurk illusion for the male/female incongruent stimuli used in this experiment.

Factorial ANOVAs are very commonly used in psychology, and we assume that many readers will be familiar with these tests, which in part motivated our choice to include this test. However, ANOVA assumes that residual errors are normally distributed, with equal variances for the different conditions, but counts of fusion percepts are natural numbers that may be better modelled with binomial, rather than normal, distributions. As an additional post-hoc test, we therefore chose to perform sign-tests to compare numbers of fusion percepts before and after familiarization and priming. This allowed us to confirm the results of the ANOVA using a non-parametric approach which is insensitive to violations of the normality assumption.

To guard against inadvertent p-hacking, the data analysis methodology was chosen prior to data collection, and the data were only analyzed after data collection was completed.

### 2.4. Participants

**Figure 2:**
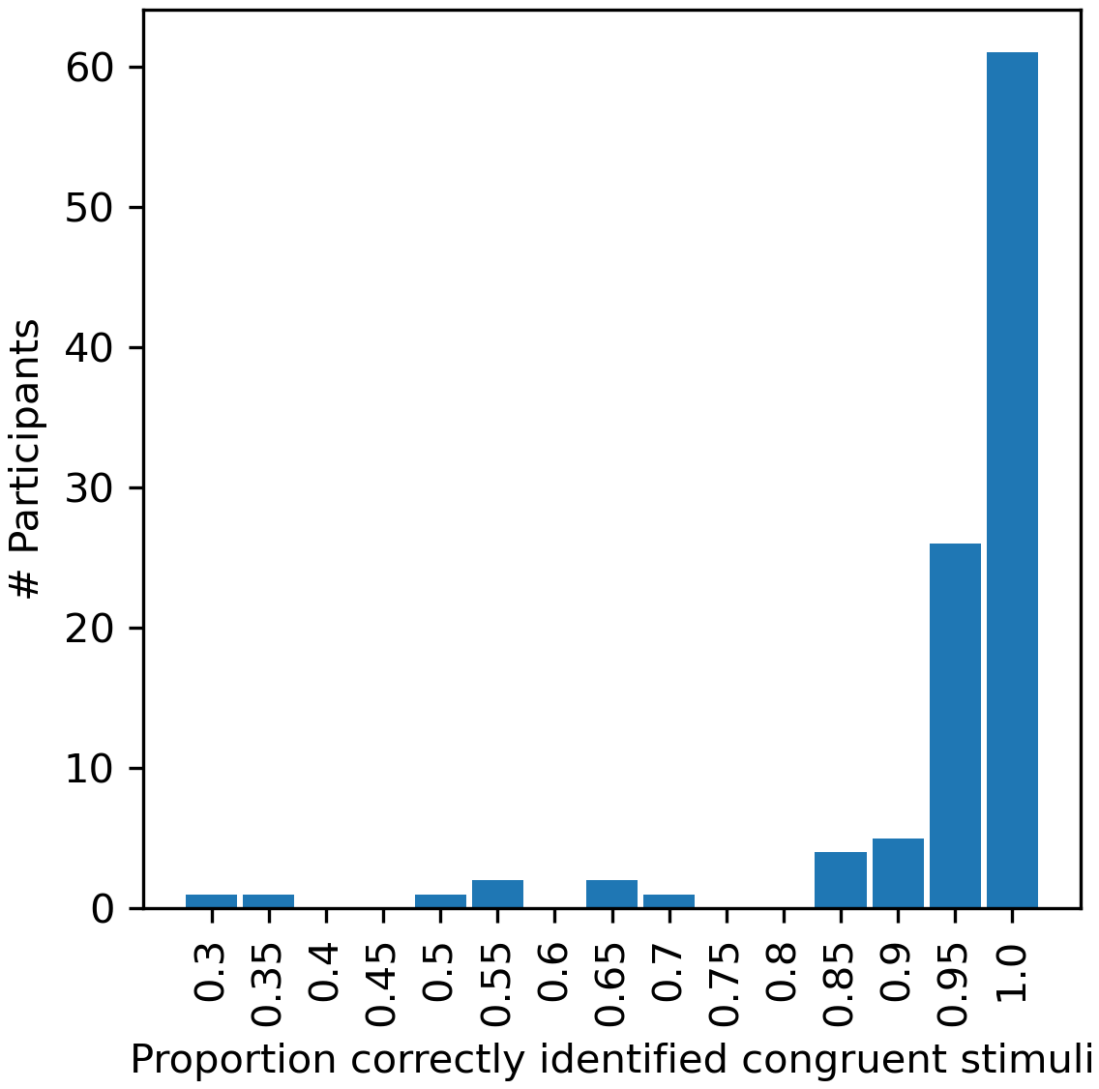
Proportion of AV-matched trials in which the syllables were correctly identified by each candidate. Correct identification of such syllables ought to be fairly easy, and is a necessary precondition for the experiment. Data from participants achieving <85% correct (pooled over Blocks 1 and 3) were therefore excluded from further analysis.

Rather than performing *a priori* power calculations to determine the number of participants to recruit, we decided instead to try to maximize statistical power by recruiting as many participants as we could within our time and resource constraints. One hundred and sixty-nine participants were recruited from online platforms, including the Open University’s student forum, the McGill University Auditory List, social media, as well as word of mouth. Participants took part in the experiment anonymously and received no payment or other incentives. The only factors motivating the participants were their curiosity and their willingness to help anonymously. Given the lack of other incentive or compensation, and given that the experiment required non-trivial amounts of time and concentration, it is perhaps surprising that as many as 104 of the 169 participants who started the experiment completed it, whilst only 65 participants (38%) dropped out before completing the experiment. Another 15 participants were excluded for failing to report the correct syllables in the control audio only trials or failing to perform at a high level (>85% correct) for matched AV trials (compare Figure 2). The identification of AV matched syllables should be an easy task, and low performance on this aspect of the task indicates that the participant was unable to perform the task properly, perhaps due to technical difficulties with their individual setup, or poor attention or motivation. A minimum 85% correct exclusion criterion was therefore used to ensure that only participants capable of identifying AV matched syllables with reasonably high accuracy were included in the final analysis. Note that analyzing the data as described above after applying stricter exclusion criteria of 90 and 95% correct (raising the number of excluded participants to 18 or 42 respectively) showed the same, significant effects (data not shown). Of the remaining 89 participants, 10 did not report their age or gender, but were included for all analyses except for the age range calculation (ages 18 to 62 years, mean 36.85, SD 11.75) or gender counting (63 females and 25 males).

The 89 participants whose data were included in the analysis differed somewhat in the amount of time included in the experiment, and the distribution of time taking had a long positive tail, indicating that a small proportion of the participants availed themselves of an opportunity to take breaks. The 5^th^, 25^th^, 50^th^, 75^th^, and 95^th^ percentile of the time taken were 17.5, 21.6, 24.4, 29.0, and 72.5 min, respectively.

All participants reported normal or corrected-to-normal vision and normal hearing, and were naïve regarding the specific aims of the study before debriefing.

## 3. Results

### 3.1. A highly significant effect on fusion responses

**Figure 3:**
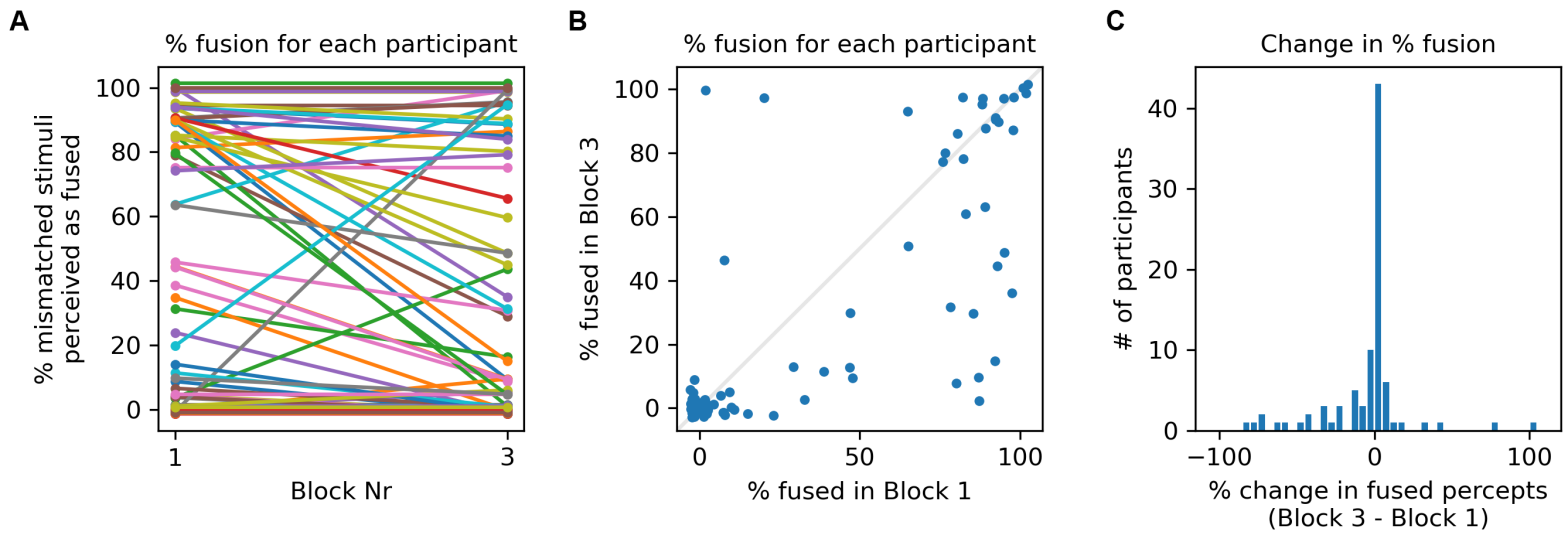
**(A)** Each line connects the % of fusion percepts reported in response to male/female incongruent stimuli by one participant in Blocks 1 and 3, respectively. Note that there are more participants for which the percentage of fusion percepts declines than there are participants for which if increases. For display purposes only, a small random jitter (drawn uniformly from a +/−3% range) was added to the y-coordinate, so as to spread out otherwise overlapping data points. **(B)** Same data as in **(A)** shown as a scatter plot. Each point represents one participant. The x- and y-coordinates show the percentage of fusion percepts they reported in Blocks 1 and 3, respectively. There are more data points below than above the main diagonal, indicating that a decline in percentage fused percepts was more common than an increase. Here too, independent, uniformly distributed jitter from a +/−3 range was added to the coordinates to spread out overlapping data points. **(C)** Distribution of changes in percentage fusion percepts between Block 1 and 3 for all participants. The trend for fusion percepts to decline is visible as a fat left tail in the distribution.

Figure 3A shows the proportion of AV mismatched, male/female incongruent stimuli that gave rise to McGurk fusion percepts in Blocks 1 and 3, respectively for all participants. Lines of different colors connect the proportions of fusion percepts in each block for each participant. Several features are noteworthy: first, participants differed greatly in the proportion of fusions perceived, exhibiting the full range, from 0 to 100% fused percepts. Second, while for many participants the proportion of fused percepts hardly changed between Blocks 1 and 3, others exhibited large changes. Third, decreases in the proportion of fusion percepts appeared to be more frequent than increases. Figure 3B shows the same data as a scatter plot, with one dot per participant, and the x-and y-coordinates showing the percentage of fusion percepts they reported in Blocks 1 and 3, respectively. Note that there are more points below the main diagonal (shown in light gray) than above it, which indicates that reductions in percentage fusion percepts were more common than increases. These observations are confirmed in Figure 3C, which shows the distribution of changes in percentage fusion percepts (Block 3 minus Block 1) in histogram form. In 34 cases, the number of fusion percepts decreased, compared to 12 increases and 43 cases of zero change. The histogram thus has a heavier negative tail.

An analysis of the data using a 2 × 2 repeated measure factorial ANOVA confirmed significant effects, both for “AV match” [*F*(1, 88) = 57.57, *p* < 0.001, np^2^ = 0.395], and for “Block” [*F*(1, 88) = 5.27, *p* = 0.024, np^2^ = 0.056], as well as a significant interaction between “AV match” and “Block” [*F*(1, 88) = 7.1, *p* = 0.009, np^2^ = 0.075]. The significant “AV match” effect confirms that it was common for our participants to experience a McGurk illusion, that is, they perceived significantly more fusion percepts /da/ when presented with discrepant visual and acoustic inputs (mean = 6.72, SD = 8.1), than when presented with matched AV pairings (mean = 0.09, SD = 0.33). The significant effect of “Block” shows that, after familiarization in Block 2, participants heard overall significantly fewer fusion percepts /da/ (mean = 5.35; SD = 7.62), compared to the reported fusion percepts before familiarization and priming (mean = 6.72; SD = 8.1). This is not unexpected if we assume that the familiarization and priming can disrupt the McGurk effect. Finally, the significant interaction between “AV match” and “Block” [*F*(1, 88) = 7.1, *p* = 0.009, np^2^ = 0.075] shows that participants indeed reported fewer fusion percepts /da/ for the AV mismatched stimuli after familiarization and priming, giving strong and direct evidence in support of the main hypothesis of this study.

**Figure 4:**
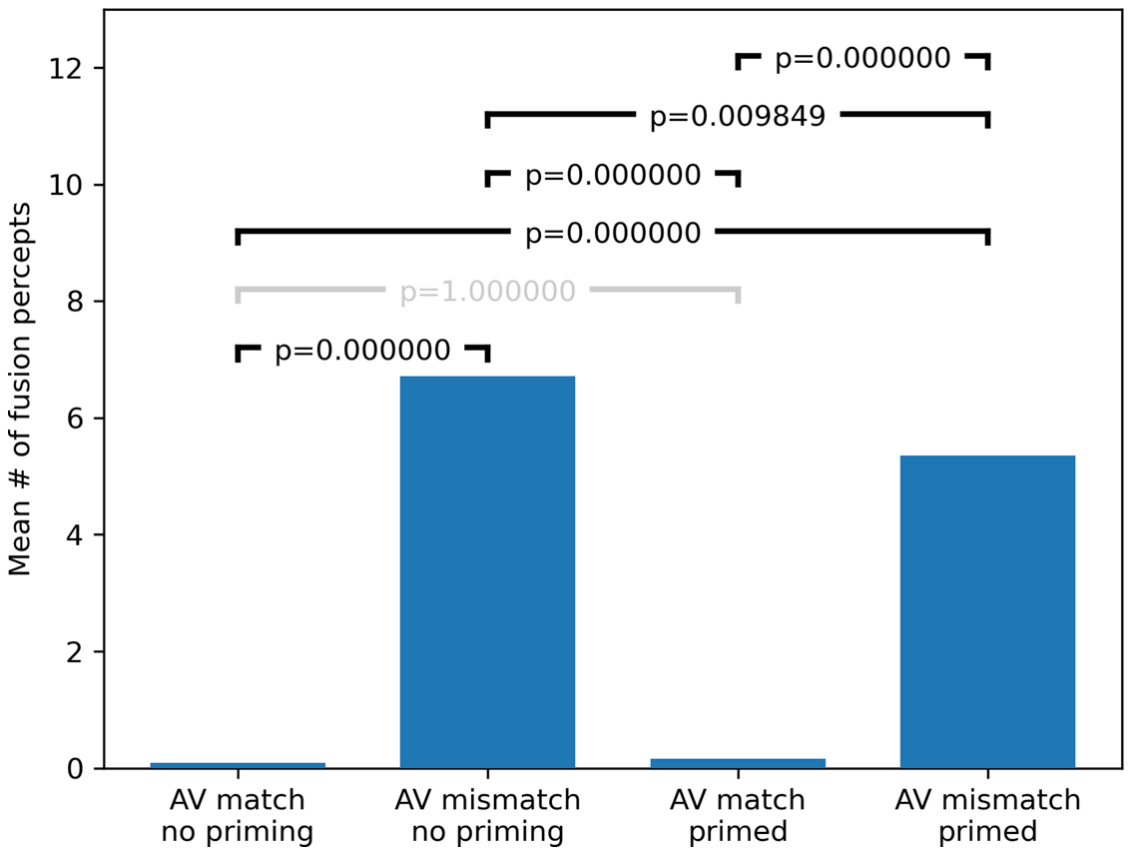
Bonferroni corrected *post hoc* test results. *p*-values were computed with pairwise sign tests comparing each condition and Bonferroni corrected by multiplying by 6 (the number of multiple comparisons) and capping at 1 (to keep corrected probabilities in the interval [0, 1]). Corrected *p*-values are shown in the brackets above the bars which indicate mean fusion percept counts across the whole cohort in each condition. Gray brackets denote non-significant differences.

Post-hoc sign tests (Bonferroni corrected) revealed highly significant differences in the number of observed fusion percepts for all (six) pairwise comparisons except for AV matched stimuli before and after familiarization and priming (*p* = 1), as these were close to zero at the outset and did not significantly change with familiarization and priming. The results of the *post hoc* tests are summarized in Figure 4. Of particular relevance is the comparison in the number of fusion responses for AV mismatched stimuli before and after familiarization and priming, where we observe a statistically significant reduction (*p* = 0.0098), which also confirms the hypothesis that familiarization and priming can disrupt the McGurk effect for male/female incongruent stimuli.

The participants in our study were randomly allocated to two groups, one presented a male face with a female voice in Blocks 1 and 3, and one presented with a female face and a male voice. It is well known that different face/voice pairings can differ greatly in how likely they are to evoke McGurk illusions. This appears to be the case here too: of the 89 participants who concluded the study, 45 participants were tested with the male face, and 36 of them reported experiencing McGurk illusions during at least some of the trials in Block 1. The proportion of fusions reported by these 36 participants declined on average by 17% in Block 3. In contrast, of the 44 participants tested with the female face, only 13 reported any McGurk illusions during Block 1. For these, the proportion of fusions reported in Block 3 declined on average by 10%. Thus, we observed considerable differences in baseline rates of fusion percepts as a function of the particular stimulus combination used, but the effects of familiarization and priming were consistent for both of the face/voice combinations tested.

### 3.2. No effect on visual dominance responses

Our outcome measure so far was the number of fusion /da/ responses to male/female incongruent A: /ba/ V: /ga/ stimuli reported by our participants before and after familiarization and priming. This outcome measure was chosen because hearing a fusion percept is the most common form of visual influence observed when observers were presented with an A: /ba/ V: /ga/ stimulus, and a count of fusion percepts is very commonly used in this type of research. However, the multisensory interactions in these types of tasks can also occasionally manifest as a “visual dominance”: participants presented with A: /ba/ V: /ga/ may report that they perceived a /ga/ if lipreading dominated on a given trial. In our dataset, we observed a total of 148 /ga/ responses to the male/female incongruent A: /ba/ V: /ga/ stimuli, considerably less than the 770 /da/ responses to the same stimulus. Therefore, while fusion was by far the most common type of visual influence, it was not the only one observed.

It is not immediately obvious whether visual dominance responses should be considered as essentially qualitatively similar to fusion responses, and whether perhaps both types of response should be summed to compute a total AV interaction score in the analysis of our data. The fact that visually dominant responses were relatively rare though (a mere 5.4% of the total of 3,548 responses to AV mismatched stimuli logged) makes this question difficult to answer authoritatively. While the number of fusion responses did significantly decline after familiarization and priming, from 598 to 476 (*p* = 0.0016, sign test), the number of visual dominance responses showed a small, statistically insignificant increase (from 93 to 98, *p* = 0.37, sign test). Adding visual dominance responses to fusion responses to calculate total AV interactions and analyzing the total AV interactions score with the same ANOVA we performed above for fusion responses leads to identical conclusions as performing the ANOVA on fusion responses alone: we still observe significant effects, both for “AV match” [*F*(1, 88) = 11.37, *p* < 0.001, np^2^ = 0.114], and for “Block” [*F*(1, 88) = 5.85, *p* = 0.018, np^2^ = 0.062], as well as a significant interaction between “AV match” and “Block” [*F*(1, 88) = 7.72, *p* = 0.007, np^2^ = 0.081]. In summary, we observed, firstly, that visual dominance responses to male/female incongruent AV mismatched stimuli were rare, making them hard to study unless one can collect very large amounts of data, secondly, that, in our dataset, familiarization and priming had no significant effect on the number of visual dominance responses observed, and third, if we add the small number of visual dominance responses to the fusion responses results to compute a total AV interaction score, the trends in the relatively much more common fusion responses dominate and the overall conclusions do not change.

### 3.3. No effect of performance in the familiarization task (Block 2)

**Figure 5:**
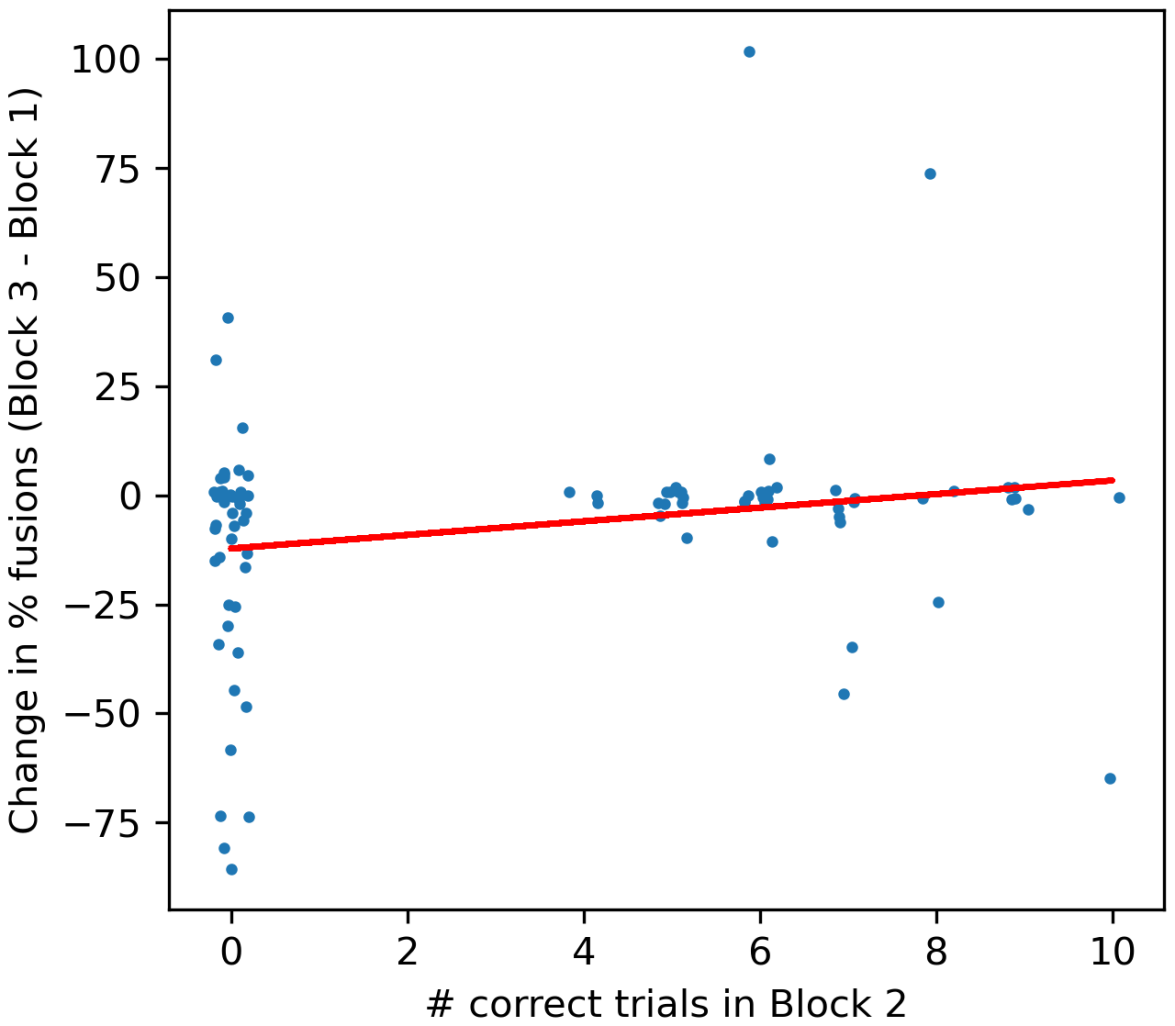
Scatterplot of number of correct responses in Block 2 against the change in the proportion of fusion responses from Block 1 to Block 3. A uniform random jitter with range +/− 0.2 was added to the x-coordinates and a jitter with range +/− 2 was added to the y-coordinates in order to spread out overlapping data points. Each dot gives the data for one participant. The red line is a linear regression line. The regression slope is not statistically different from zero (R^2^ = 0.04, *F* = 3.7, df = 87, *p* = 0.06), and it does slope in the opposite direction from that expected if high scores in Block 2 were necessary to experience decreases in the number of fusion percepts in Block 3.

As described above, the task in Block 2 was designed to facilitate the formation of associations between the “true,” congruent pairings of faces and voices by presenting two faces side by side (one of which the original face from Block 1), which both articulated syllables /ba/, /ga/ or/ da/ with their actual voices at random, potentially overlapping time intervals, and requiring the participants to count the number of times the face encountered in Block 1 articulates one of these syllables. This task is very difficult, making high demands on selective attention as well as working memory, and this is borne out by the performance of our participants. The highest score obtained by any of the participants was 10 out of 17 trials correct, and almost half (44/89) participants scored not a single trial correct. Making the task difficult was a deliberate design choice. Paying selective attention to one of the two competing speakers should only become easier once the correct pairing of voice and face becomes well established in the participants’ minds, and making the selective attention task difficult might engage attention more fully, which in turn might facilitate the formation of the correct associations. However, good performance on this task depends on many factors, such as a long attention span and a strong working memory to keep count, which have little or nothing to do with the McGurk illusion, and we therefore did not expect the scores in Block 2 to be strongly predictive of changes in number of reported McGurk illusions between Block 1 and Block 3. Even participants who scored very poorly in Block 2 will have had ample opportunity to familiarize themselves with the correct face-voice pairings merely by watching the Block 2 videos, in which the synchronization of audio and video streams, as well as the male/female congruence between voice and face. This provided an abundance of cues about which voice really belongs to which face. Figure 5 shows the change in the proportion of reported fusion percepts against the number of correct responses in Block 2, and indeed, we observe no significant relationship.

### 3.4. No effect of priming syllable type

In Block 3, we used two different matched AV syllables as primes, either /ba/ or /ga/, presented in pseudo random order. This raises the possibility that the type of syllable presented might introduce a confound. A previous study has shown that the McGurk illusion can be stronger if the AV syllable presented is shown several times in rapid succession, rather than just once (Magnotti et al., 2018). While in our Block 3 the situation is somewhat different, in that we do not repeat the same mismatched AV syllable more than once, we nevertheless need to be mindful of the possibility that presenting either /ba/ or /ga/ as primes, respectively, could potentially create different contexts which might influence the outcome. To verify that this was not the case, we computed the percentage of fused responses for /ba/ −primed and /ga/ −primed trials in Block 3 respectively, and used Wilcoxon tests to compare these against each other, as well as against the percentage of fused responses in Block 1. We found that the difference in percentage fused responses for /ba/ −primed trials (mean 27.6) and /ga/ −primed trials (mean 25.8) fell just short of statistical significance (*p* = 0.06). However, both were highly significantly smaller than the percentage of fused responses in Block 1 (mean 33.8, *p* = 0.0014 and 0.0007 respectively). Thus any potential effect of the type of priming syllable chosen appears to be too small to confound the overall result of this experiment.

## 4. Discussion

This study found a significant reduction in the susceptibility to the McGurk illusion in male/female incongruent stimuli after priming a face with a congruent, same-sex voice. Before familiarization and priming, some participants frequently perceived the fusion percept /da/ when presented with discrepant visual /ga/ and acoustic /ba/, and reported the acoustic syllables correctly in most audio-visual matched trials. After familiarization and priming, these participants heard fewer fusion percept /da/ when presented with discrepant audio-visual inputs. Our data also revealed considerable inter-subject variation, both in how strongly they perceived the McGurk illusion in the first place, and how strongly they are influenced by familiarization and priming. However, it is perhaps remarkable that a statistically significant effect can nevertheless be observed after as little as ~10 min of familiarization training and subsequent priming.

It is important to acknowledge that this study has a number of limitations, which stem both from the fact that the experiment was conducted at a time when COVID related lockdowns precluded bringing participants to the lab for testing under highly controlled conditions, and from the fact that the study was performed without any budget, and resourced only with the time, effort and access to personal equipment that the authors and unremunerated, anonymous volunteer participants were prepared to contribute. In designing the study, we therefore had to make a number of compromises. The experiment had to be short, so as not to overtax the goodwill of our participants, and this precluded the inclusion of additional control conditions which would have been desirable to further confirm or differentiate the effects we observed. Consequently, whether familiarization training and priming are both needed to produce such rapid changes, and which of these has a stronger effect remains currently unknown. We also do not know whether familiarization might have an effect even if there is no male/female incongruence in the stimuli used. Similarly, it might have been desirable to test a larger set of face and voice stimulus combinations to demonstrate that the effect generalizes beyond the two male/female face permutations tested, or to incorporate additional stimuli which might try to produce an independent measure of how strongly voices and faces have become associated at the end of Block 2, and to see whether this strength of association correlates with observed reductions in the McGurk effect. And like most other studies of the McGurk effect, this study also suffers from a degree of uncertainty about whether the participants maintain an even allocation of attention to the visual and auditory modalities throughout.

Despite these caveats, we have a relatively high confidence in our results, in part because our analysis is very conservative and the observed effect is quite large, and in part because our results are in good agreement with those described in other, related studies. For example, relatively rapid modulations of the likelihood that observers perceive McGurk stimuli as fused have been observed before. The study by Gau and Noppeney (2016) mentioned in the introduction created AV-matched or mismatched stimuli from recordings of one female speaker, and then manipulated context by embedding McGurk stimuli in blocks of stimuli which were either AV matched, or so mismatched as to be impossible to fuse. Participants fused McGurk stimuli more readily when these were presented in an AV matched context, where AV fusion is very easy and “correct,” than in blocks where frequent, profound AV mismatch made attempts of fusion seem futile. This suggests that the participants’ expectations regarding whether or not an AV stimulus is likely fusible influences how easily a McGurk illusion is perceived, and these expectations can change relatively quickly.

The earlier work by Green et al. (1991) and Walker et al. (1995), which inspired our study used male/female incongruent stimuli to challenge a participants’ expectations on the basis of their prior knowledge and experience, unlike Gau and Noppeney (2016), who relied purely on the local context provided by varying degrees of AV mismatch in each block to alter multimodal perception without introducing male/female incongruence as a variable. Our study followed in the footsteps of Walker et al. (1995), but asked whether the interplay of male/female incongruence effects and familiarity with particular voices and faces that they had observed could be established very rapidly, in a manner of minutes. In line with our hypothesis, we observed that familiarization and priming reduced the number of fusion percepts specifically for the discrepant A: /ba/ V: /ga/ pairings, but not for phonetically matched AV inputs. Bear in mind though that the number of fusions for the matched inputs was close to zero, as expected, so large reductions would have been impossible. After knowing a particular voice belongs to a particular face, participants were on average less likely to form assumptions of unity when that same voice was dubbed with an incongruent, opposite-sex face. Familiarization and priming here served as a short-term context cue that strongly reminded participants that a particular voice belongs to a particular face, and thus a less strong assumption of unity was formed when the same voice was dubbed with an incongruent, opposite-sex face. The present study thus gives another example of knowledge and experience (i.e., top-down factors) influencing multisensory integration and modulating intersensory bias in humans speech perception, and these findings suggested that immediate perceptual response to intersensory discrepancy can be induced or reversed by familiarization and priming.

However, note that not all participants experienced such a familiarization and priming effect. Indeed, Figure 3B shows that for many (43 out of 89) participants, the number of fusion percepts did not change at all, and for 12 it went up, compared to 34 for whom it went down. Thus, even though there was a robustly significant effect of familiarization and priming on average of the whole cohort, for a substantial number of participants the McGurk effect remained unchanged even after they had been both trained and primed just seconds earlier that the voice was not congruent with the face. Also noteworthy is that 36 participants, about 40% of the total, did not experience a single McGurk illusion during the entire experiment. Individual differences are therefore very substantial. Nevertheless, both the non-parametric sign-test and the ANOVA performed gave a highly significant result, providing strong evidence in support of the hypothesis that familiarization and priming on average reduce the McGurk effect with male/female incongruent stimuli, considerable subject-to-subject variability in the susceptibility to and malleability of the McGurk illusion notwithstanding.

This study was partly inspired by Green et al. (1991), who asked their participants to watch clips containing discrepant phonetic information that were either male/female congruent or incongruent, and found that participants still integrated the auditory and visual inputs into a single percept regardless of the obvious male/female incongruence between the voice and face. The result by Green et al. (1991) is on some level rather surprising, given that causal observers cannot fail to notice that the voice and face are clearly mismatched, but also because other studies have shown that male/female incongruence can indeed disrupt aspects of multisensory integration. For example, Vatakis and Spence (2007) presented participants with AV speech stimuli in which the onset of the auditory and visual stimuli were deliberately desynchronized, and asked the participants to judge whether the sound or the lip movement started earlier. They observed that participants performed less well at this task if the AV syllables presented in this task were male/female congruent than when they were incongruent, which they interpreted to mean that the congruent AV syllables were more strongly perceived as a unit than the incongruent ones, which makes the question “which comes first” harder to answer. While our study supports Green et al.’s observation that McGurk illusions are common even if stimuli are male/female incongruent, it also demonstrates that McGurk illusions with male/female incongruent stimuli are on average reduced after familiarization and priming. This reduction in McGurk illusions is partly at odds with Green et al.’s conclusion that the male/female incongruent McGurk effect falsifies Welch and Warren (1980) unity assumption hypothesis.

Welch and Warren (1980) theorized that intersensory bias formed only under certain conditions, which include “temporal synchrony” and “general experience.” In the McGurk illusion, the temporal synchrony between seen lip movements and heard audio is thought to create a “highly compelling” situation, in which the assumption of unity is very strong. However, adult males typically have longer vocal tracts than females. Male voices are therefore characterized not just by a generally lower voice pitch (which could be disguised if an individual chooses to change their voice pitch by altering the tension on their vocal folds), but also by lower formant resonance frequencies (over which speakers have no voluntary control as they can do little to shorten or lengthen the distance from their vocal folds to their lips and noses). “General experience” sensitizes human observers during childhood to the pronounced sexual dimorphism in these acoustic cues, and to distinguish easily and accurately between typical male and female voices (Nagels et al., 2020). Most observers will therefore experience a strong incongruence if a male looking face appears to speak with a female sounding voice. This incongruence might be expected to conflict with the idea of a “highly compelling” situation stipulated by Welch and Warren (1980) and Green et al. (1991) therefore interpreted the existence of male/female incongruent McGurk illusions as evidence against Welch and Warren’s theoretical framework, which posited the importance of top-down influences shaped by experience in fusing sensory data within and across modalities into unitary events (Welch and Warren, 1980). A wealth of research has manipulated various factors that may lead to different levels of the intersensory bias and demonstrated that the unity assumption can be induced explicitly by experimenter’s instruction (Warren et al., 1981), or evoked by stimuli with highly congruent properties and characteristics (Chen and Spence, 2017), temporal synchrony (Munhall et al., 1996), or when perceivers have learned to associate multimodal inputs from prior experience, either long-term knowledge (Doehrmann and Naumer, 2008) or short-term contextual cues (Nahorna et al., 2015). Conversely, whether or not AV stimuli are perceived as unified can strongly affect their perceptual attributes, not just aspects like phoneme identity, which we have discussed at length, but also perceived spatial location (Wallace et al., 2004).

However, Green et al.’s (1991) objection may simply underestimate how strongly human perception weights temporal synchrony as a binding cue, and therefore the quality and quantity of “general experience” that might be required to overrule it. Synchrony is such a powerful cue in the auditory domain that it can bind frequency components presented to opposite ears into a single harmonic sound (Beerends and Houtsma, 1986), or powerfully override evidence for spatial disparity between visual and auditory sources in the “ventriloquism effect” (Warren et al., 1981). Thus, large amounts of highly specific “general experience” may be needed to overrule powerful temporal synchronicity cues which favor perceptual unity.

Walker et al. (1995) were the first to show that this may be the case, by demonstrating that male/female incongruent McGurk illusions are less likely to be observed when participants were familiar with the faces used in the construction of the mismatched stimuli, and the experience told them that the voice and face were incongruent therefore went beyond just a general sense that the voice was somehow incongruent given the sexual characteristics of the face. The effect was modest, with the mean percentage of fusion percepts for male/female incongruent stimuli declining from 28% for unfamiliar faces to 21% for familiar faces, but these numbers are not very different from those observed here, with 34% of incongruent, mismatched stimuli evoking fusion percepts before familiarization and priming, and this number declining to 27% after familiarization and priming. The present study thus similarly concludes that the susceptibility of McGurk effect in male/female incongruent stimuli will on average decrease if participants are trained to dissociate a face from an incongruent voice and are reminded of the incongruence through priming. Like the study by Walker et al. (1995), our results therefore support Welch and Warren’s theory of intersensory interactions (Welch and Warren, 1980) overall, but the large individual differences and the relatively modest overall effect size we observed nevertheless point to the fact that top-down influences are at times perhaps surprisingly weak compared to the very strong bottom-up perceptual binding cues that are created by temporal synchrony.

As well as supporting the theory of Welch and Warren (1980), our study has gone some way to overcome the methodological shortcomings of previous research. In the original study of McGurk and MacDonald (1976), 98% of adult participants reported hearing the fused percept of /d/ when a visual bilabial phoneme /g/ dubbing with an auditory velar phoneme /b/, but later studies found a high degree of variability across individuals (Mallick et al., 2015), and this variability may interact with the specifics of the video and audio clips used to construct the stimuli (Stropahl et al., 2017). By using a within-participants design in which standardized amounts of experience were provided to familiarize participants with the correct (congruent) face-voice pairings, individual differences could be excluded as a potential confound. Also, by crowdsourcing data collection on the internet, this study was able to recruit a comparatively large number of participants. The study by Walker et al. (1995) had 18 participants in their experimental group and 18 in the control group. Our study, with 89 participants tested in both the control and the experimental conditions, has greater statistical power than any previous exploration of this phenomenon.

This study also differs from most other previous studies on the McGurk effect, in that the experiment was conducted entirely online, although it is not the first to do so (Mallick et al., 2015; Getz and Toscano, 2021). Indeed, Magnotti et al. (2018) have previously compared data from McGurk experiments conducted online and in person, and found no significant differences in the data obtained in either condition. This may no doubt add to the variance in the data, given that each participant used their individual equipment in a place of their own choosing, which precludes careful calibration and standardization of listening conditions. We had no means for monitoring participants’ eye positions, and one cannot be certain to what extent participants followed the instruction to fixate on the speaker’s face. This is unlikely to be a big problem though, given that, the proportions of McGurk illusions we observed are comparable to those reported in previous studies, and this could not have been the case if a high proportion of our candidates had experienced difficulties observing the video clips. Similarly, the use of AV-matched control stimuli throughout Blocks 1 and 3 allowed us to ensure that only data from participants who were able to attend to acoustic stimuli without undue amounts of distraction or disturbance from an inadequate audio quality were included in the analysis. Furthermore, by choosing a within-participant design, we were able to ensure that the increase in intra-subject variance that is likely to arise from the lack of standardization and control of the listening conditions did distribute equally across control and treatment conditions. Thus, while for many types of psychophysical experiments, very precisely calibrated and carefully controlled conditions will no doubt remain essential, for other types of experiments, the current one included, conducting an online experiment which can ensure that the observed effects are observable “in the field,” and not just under highly specific laboratory conditions, can no doubt be advantageous, and it is perhaps unsurprising that online experiments, while still a relatively small minority, are rapidly gaining in popularity and acceptance (Gosling and Mason, 2015; Woods et al., 2015; Getz and Toscano, 2021).

Another advantage of conducting experiments online is that it allows testing of potentially significantly larger cohorts. Even though we had no budget to offer a financial incentive to our participants, we were able to recruit a sizable cohort, with 169 participants trying out our experiment, and 104 completing it, motivated simply by curiosity and a desire to be helpful. Better resourced online experiments can leverage crowdsourcing websites such as Amazon mechanical turk to recruit several hundred participants into an experiment and test them in parallel (McPherson and McDermott, 2018), which opens up possibilities that are unattainable in a classic laboratory setting. That said, we must also acknowledge the downsides of this particular online experiment. Since we were unable to offer remuneration to our volunteer participants we were dependent on their goodwill, which in turn created pressure to keep the duration of the entire experiment unusually short so as to avoid exceedingly high dropout rates. Including briefing, practice tests, the 3 experimental blocks and debriefing, most participants were able to complete the whole experiment in under half an hour. This pressure to keep the experiment short precluded us from adding additional stimulus conditions that might have been interesting to try. Having tested only one pair of faces and voices on each participant, we are unable to gauge the extent to which our results might generalize to other faces, syllables, or stimulus conditions. Similarly, disambiguating the relative contributions of the familiarization block and the priming, something that would in principle be easy to assess if participants can be tested for longer, will have to await future studies. Nevertheless, this work demonstrates that even very low budget online experiments can make contributions to the field of sensory psychology.

Overall, this study complements previous studies which support the unity assumption hypothesis, providing a clear example of top-down modulation of human multisensory perception. Follow-on studies could map out the relative weighting of bottom-up synchronicity cues versus top-down influences of incongruence or experience by testing whether decreasing the precision of the temporal synchrony between audio and video clips allows for stronger top-down effects.

## Data availability statement

The original contributions presented in the study are publicly available. This data can be found here: https://osf.io/sc4uj/.

## Ethics statement

The studies involving humans were approved by DE300 ethical review board of the Open University. The studies were conducted in accordance with the local legislation and institutional requirements. The participants provided their written informed consent to participate in this study. Written informed consent was obtained from the individual(s) for the publication of any potentially identifiable images or data included in this article.

## Author contributions

KM designed the experiment, collected the data, and wrote the manuscript. JS implemented the online experiment and edited the manuscript. All authors contributed to the article and approved the submitted version.

## Funding

No specific funding was available for this work. Publication costs were defrayed by an internal grant from City University to JS.

## Conflict of interest

The authors declare that the research was conducted in the absence of any commercial or financial relationships that could be construed as a potential conflict of interest.

## Publisher’s note

All claims expressed in this article are solely those of the authors and do not necessarily represent those of their affiliated organizations, or those of the publisher, the editors and the reviewers. Any product that may be evaluated in this article, or claim that may be made by its manufacturer, is not guaranteed or endorsed by the publisher.

## Footnotes

^1^https://ffmpeg.org/
